# In-vitro study of the implant bed cooling during guided implantation using an additively manufactured drilling template with an integrated cooling system

**DOI:** 10.1186/s40729-025-00614-w

**Published:** 2025-03-19

**Authors:** Vadim Kopzon, Sebastian Hahnel, Alexander Broll, Julian Fuellerer, Georg Beierlein, Martin Rosentritt

**Affiliations:** Department of Prosthodontics, University Medical Center Regensburg, Franz-Josef-Strauss-Allee 11, 93053 Regensburg, Germany

**Keywords:** Bone heating, Dental implant, Drill cooling, Printed template, Guided template

## Abstract

**Purpose:**

The aim of this study was to investigate the performance of a novel 3D-printed cooling system for drilling templates during fully guided implant insertion.

**Methods:**

Dental implant tunnel preparations were performed for the Straumann Bone Level implant in a 3D-printed synthetic resin model using either conventional guided or modified 3D-printed guided (with a cooling channel leading directly to the implantation site) drilling templates. Temperature measurements were performed with and without cooling at drill depths of 2, 4, 7, and 10 mm.

**Results:**

For all drill depths and templates, cooling had a statistically significant (p < 0.001) influence on the measured mean temperature. ANOVA and Bonferroni correction revealed that there was a statistically significant (p < 0.001) difference in the cooling efficiency of the samples cooled with all the templates in comparison with that of the samples not cooled. The maximum temperature measured with the conventional template was 35.2° without cooling and 26.6 °C with cooling at depths of 2 and 10 mm, respectively. For the modified template, the maximum temperature reached 39.1 °C without cooling and 31.2 °C with cooling at depths of 10 and 2 mm, respectively.

**Conclusions:**

Compared with the conventional cooling system, the newly developed internal cooling channel of the modified drill template did not lead to a better cooling effect.

**Graphical Abstract:**

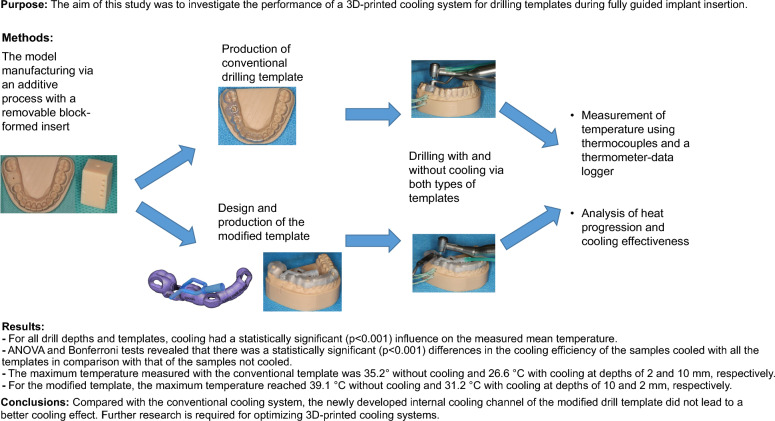

## Background

In implantology, during the preparation of an implant tunnel, bone heating is a problem that has not yet been fully resolved. Severe overheating of the bone tissue can lead to osteonecrosis, which impairs the desired osseointegration [1–3] of the implant or can even result in loss of the implant. Heating above 47 °C for 1 min is believed to disrupt bone regeneration [4]. Adequate cooling of the drilling instrument, in addition to adjusting the drilling speed, plays a crucial role in successful implantation and should not be neglected. Surgical implant planning has made great progress since the inception of 3D radiological techniques (CBCT-cone beam computed tomography). Planning the implant positing using CBCT and its transfer to the clinic with digital design (CAD—computer-aided design) and drilling templates produced by CAM (computer-aided manufacturing) processes (fully guided implantation) provides significantly improved planning reliability and more precise implantation than hand-guided templates do [5]. However, with current drilling template designs, ensuring adequate cooling of the implantation site is difficult or impossible. This occurs because the template design, combined with the sleeve used for guided implantation, prevents the cooling liquid from being directly applied to the implant drill via the surgical contra-angle handpiece. Although subtractive processes in CAD/CAM methods make it possible to produce precise fitting templates, they do not allow the production of delicate internal structures with inner lumens: such structures could, however, be used to improve the cooling of the implant drill. In addition to cost effectiveness and sustainability, additive manufacturing processes have gained popularity on the market. In addition to cost-effective and sustainable production, additive manufacturing techniques also allow individual manufacturing of dentures, models, splints and templates [6]; nevertheless, the products can be manufactured with high precision and fit [7–9]. The additive manufacturing processes also make it possible to produce small cavities and internal structures with internal lumens. Our research presents a novel design for an individual guidance system within the drilling template, which directs the coolant more specifically than conventional techniques to avoid overheating of the bone in and around the implant bed during implantation. Moreover, this design does not restrict the view of the drill or the implantation area by avoiding the need for additional external structures.

The null hypothesis was that the installation of a cooling channel within the drilling template, which guides the cooling liquid as closely as possible to the site of bone penetration, would lead to better cooling of the drill and the surrounding bone.

## Materials and methods

**Fig. 1:**
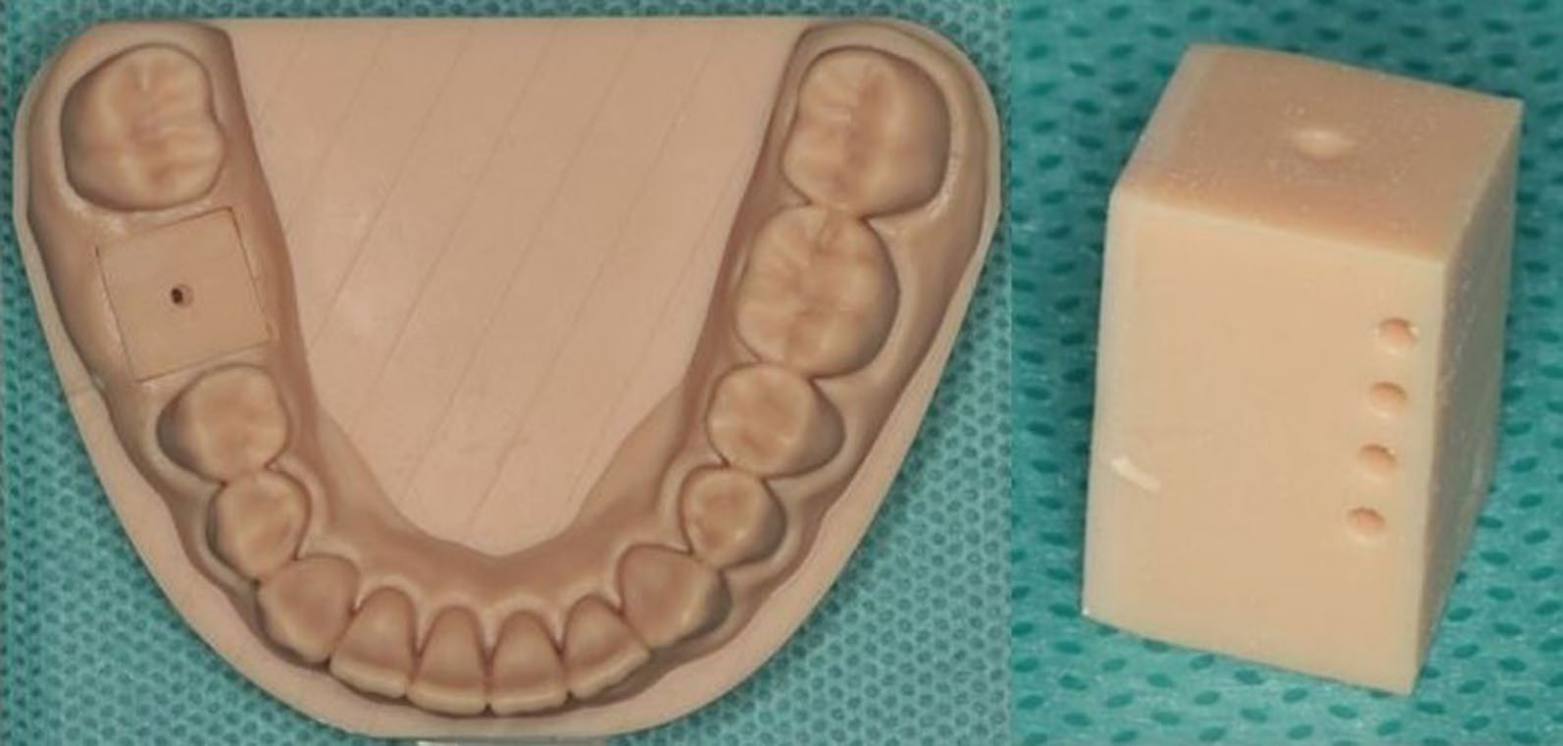
Left: Printed synthetic resin model of the lower jaw with a preconstructed tunnel site on tooth 46 (see explanations in the text); right: Detailed removable block-formed printed insert for the model with preconstructed tunnels: a single one (top) for drilling and four (lateral) for thermocouple positioning

The temperature changes in an implant bed during an implantation process with or without appropriate cooling were determined in a model experiment. For this purpose, a digital model was designed with an appropriate software application (Autodesk Fusion 360®, Autodesk Ltd., Munich, Germany), which showed a classic example of a single missing tooth at site 46 in the mandibular posterior region. The model was manufactured via an additive process with a 3D printer (Form3B + , Formlabs Ltd., Berlin, Germany) and synthetic resin (model resin v2, Formlabs Ltd., Berlin, Germany) (Fig. 1, left). To offer multiple uses of the model (to perform a series of drillings), a removable block-formed insert at site 46 was designed and, via the additive method, printed from a material identical to the material of the model (Fig. 1, right). This insert was provided with a preconstructed tunnel with a diameter of 1.2 mm to reduce the drilling time with a pilot drill.

To measure the temperature change in the material during the implantation process (with and without cooling), defined positions (2, 4, 7 and 10 mm below the alveolar ridge) for the thermocouples (K type) were built into the insert and model at a distance of 1.0 mm from the drilling tunnel. The thermocouples were placed in the above positions to measure the intraosseous temperature close to the drill. The 1.0 mm distance refers to the tunnel with the largest diameter, measured after the final preparation using a 3.5 mm drill. To improve the thermal conductivity and avoid creating an air gap between the sensor and the model, the tunnels in the model for the sensors were lubricated with petroleum jelly.

The intraosseous temperature during the implantation process was analysed using thermocouples inserted at defined distances from the planned implant tunnel and a thermometer-data logger (Testo 176T4, Testo SE & Co. KGaA, Lenzkirch, Germany). The data logger was calibrated with software provided by the manufacturer (Testo Comfort Software Basic, Program version 5.6SP6.4.213.36674, Testo SE & Co. KGaA, Lenzkirch, Germany). Measurements were performed at every second during drilling.

**Fig. 2:**
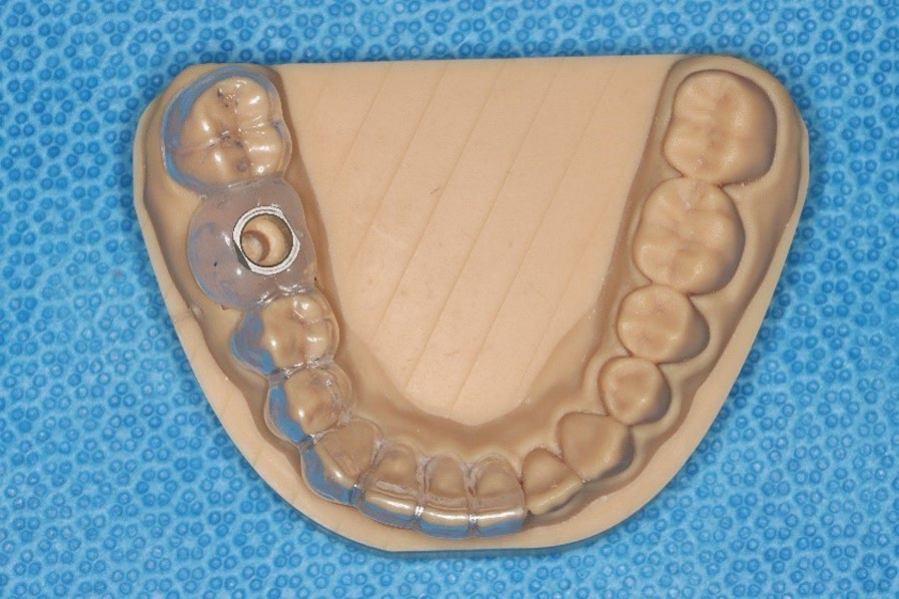
Conventional drilling template on the printed synthetic resin model of the lower jaw with an integrated sleeve at tooth site 46

A drilling template corresponding to the simulated clinical situation was subsequently constructed with a sleeve (T-sleeve, diameter 5 mm, height 5 mm; Straumann, Basel, Switzerland) for guided surgery and was conventionally produced by an experienced dental technician (Fig. 2). Approved and suitable synthetic resin materials were used (Erkodur®, Erkodent® Erich Kopp Ltd., Pfalzgrafenweiler, Germany and Palapress^®^ vario transparent, Kulzer Ltd., Hanau, Germany). The sleeve was fixed in the template with a special modelling composite (Freeform^®^, Detax Ltd. & Co KG, Ettingen, Germany).

The drilling tunnel was prepared by a single operator at the same working pressure of 1 kg (which was carried out by calibrating the operator force with a scale). A standardized drilling log for preparing an implant bed to accommodate a standardized implant (4.1 mm × 10 mm, Straumann Bone Level, Straumann, Basel, Switzerland) was carried out using a classic surgical contra-angle handpiece (Nouvag^®^ CA 16:1, Nouvag AG, Goldach, Switzerland) with constant contact pressure. The temperature change was determined for the different preparation drills (pilot drill with a diameter of 2.2 mm and speed of 800 rpm; preparation drills with a diameter of 2.8 mm and 3.5 mm and speeds of 600 rpm and 500 rpm, respectively). The standardized preparation drills were used for a maximum of 10 drillings. In the first part of the study, temperature measurements were carried out with classic cooling from a hose attached to the contra-angle handpiece, from which the coolant (0.9% NaCl, t = 23 °C – room temperature, flow rate 133 mL/min) was delivered to the drills above the template. To compare the reference values, the temperature measurements were carried out with the coolant and without (“dry preparation”). Each procedure, with and without cooling, was performed 10 times. During drilling accompanied with cooling, the cooling liquid was removed manually using a conventional dental suction device.

**Fig. 3:**
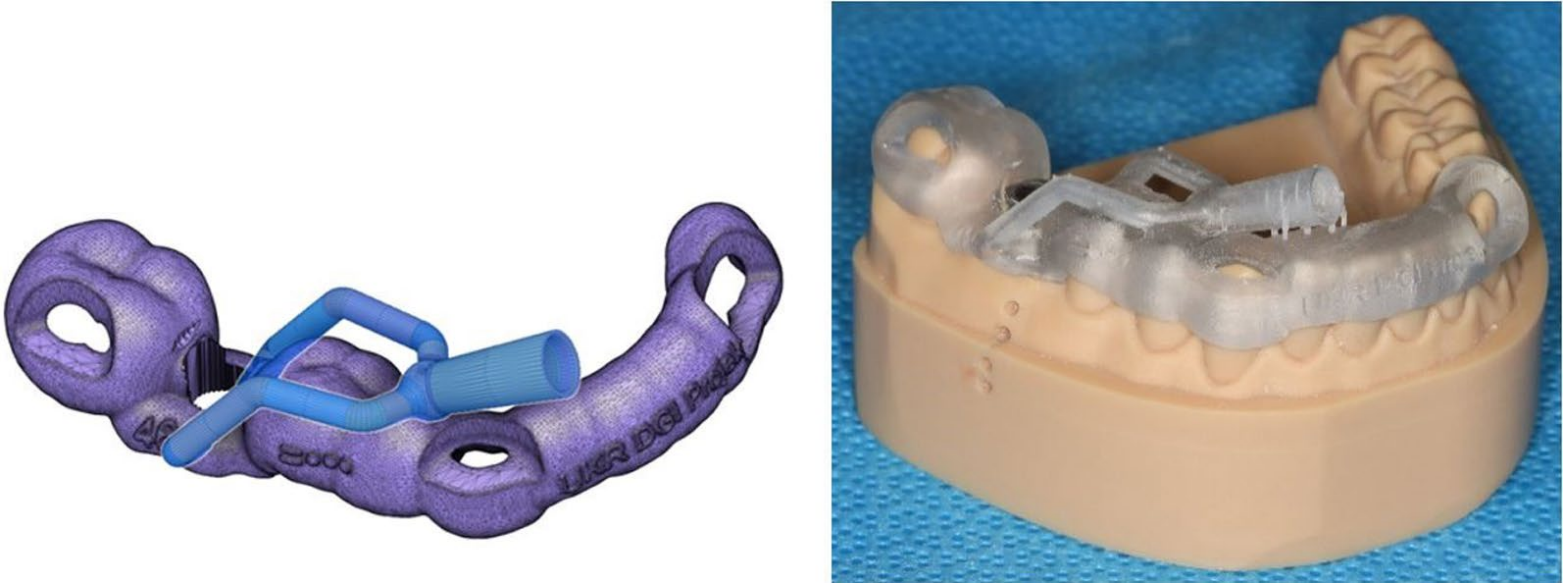
Left: modified drilling template with a cooling channel constructed via Autodesk Fusion 360^®^ (Autodesk Ltd., Munich, Germany); right: modified drilling template with an integrated sleeve at tooth site 46 on the printed synthetic resin model of the lower jaw

In the second part of the study, the experiment was carried out with a modified template. The synthetic resin model of the lower jaw was duplicated and was made from plaster and barium sulphate for radiographic contrast. CBCT (Planmeca^®^ Viso G7, Planmeca, Helsinki, Finland) was performed via the plaster model to generate a DICOM (Digital Imaging and Communications in Medicine) data set. To generate a standard tessellation language (STL) dataset, the synthetic resin model was scanned with Primescan^®^ (Primescan^®^, Dentsply Sirona Germany Ltd., Bensheim, Germany). Both datasets were processed with suitable software (coDiagnostiX®, Version 9.2 Client, Dental Wings Ltd., 09111 Chemnitz, Germany) to construct a guided drilling template for implantation at tooth site 46. The template constructed was exported as an STL dataset from coDiagnostiX® and further processed via Autodesk Fusion 360^® ^(Autodesk Ltd., Munich, Germany), in which a cooling channel leading directly to the implantation site was digitally designed (Fig. 3, left).

The final modified template was produced via a 3D printer (Form3B + , Formlabs Ltd., Berlin, Germany) with light-curing resin designed for additive manufacturing of dental drill guides (P pro Surgical Guide Clear^®^, DeltaMed Ltd., Friedberg, Germany). The sleeve for tooth site 46 was fixed into the modified template conventionally as described above (Fig. 3, right and 4).

**Fig. 4:**
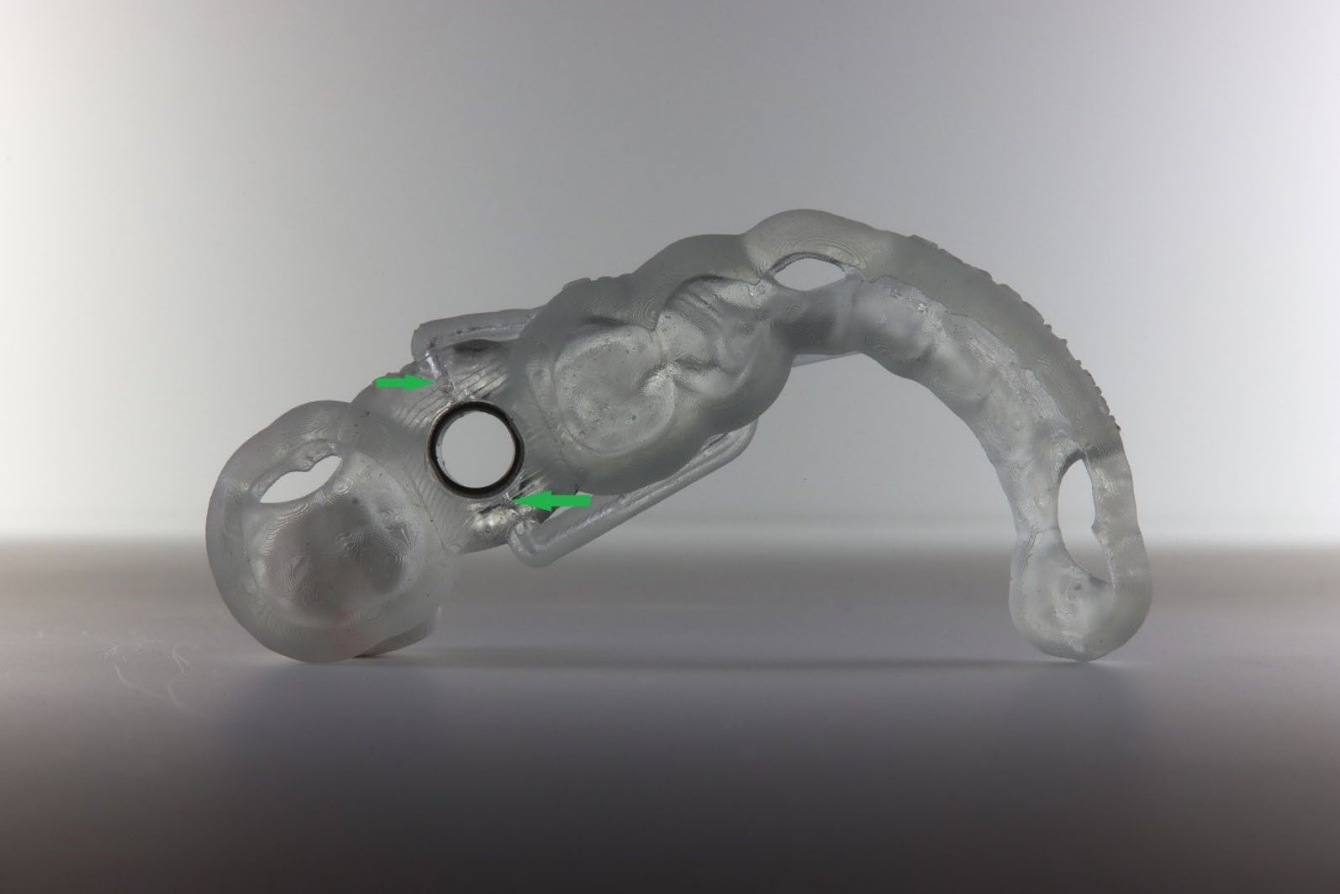
Underside view of the modified drilling template, showing cooling channel openings near the bone penetration area beneath the sleeve (green arrows)

The functioning of the cooling channels was checked after 3D printing. It was compared how much cooling liquid flows via the cooling tube of the contra-angle handpiece in 1 min with the amount of liquid that flows via the cooling system of the modified template in the same time. Both values were 133 mL/min.

**Fig. 5:**
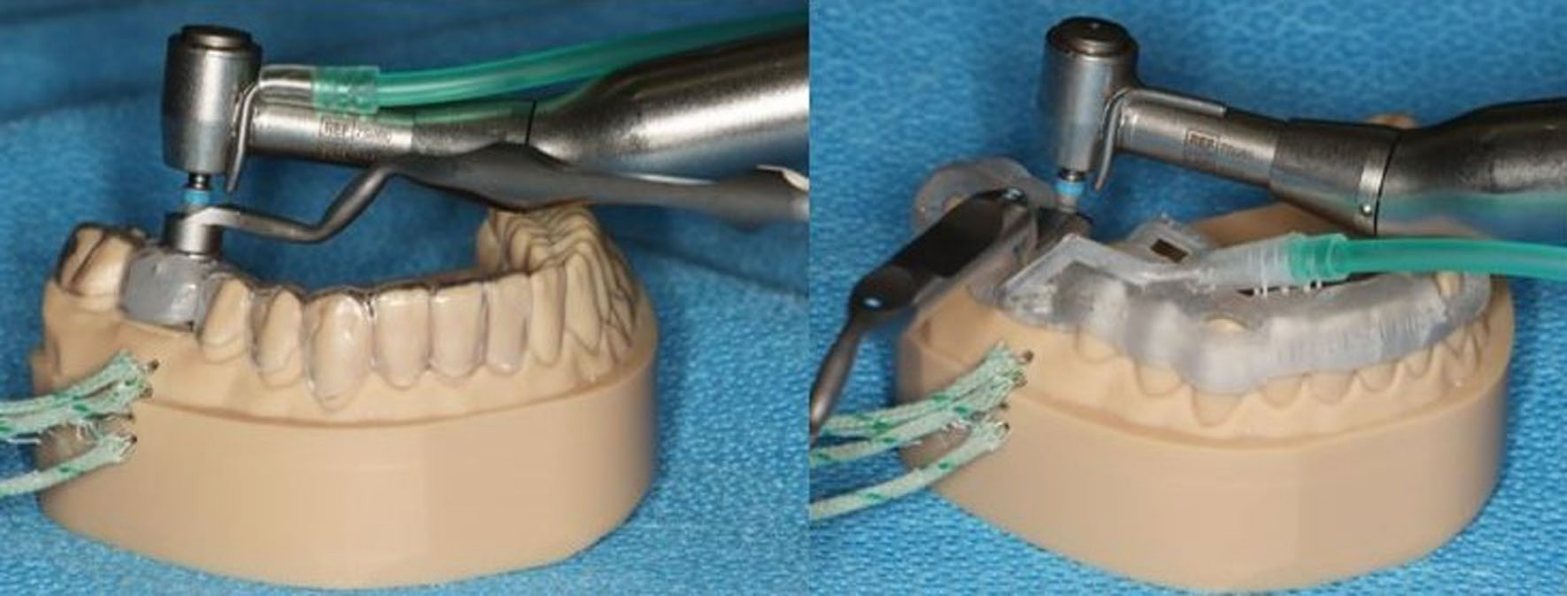
Left: Experimental setup for drilling with a pilot drill (diameter = 2.2 mm) through a conventional template, with the cooling hose attached to the contra-angle handpiece and thermocouples inserted into the side tunnels of the model; right: experimental setup for drilling with a pilot drill (diameter = 2.2 mm) through a modified drilling template, with the cooling hose attached to the innovative cooling channel system and thermocouples inserted into the side tunnels of the model

The temperature measurements were carried out by cooling from a hose attached to the cooling channel of the modified template, which delivered coolant (0.9% NaCl, t = 23 °C – room temperature, flow rate 133 mL/min) directly to the drills near the bone penetration area beneath the template. Additionally, the temperature measurements were carried out without cooling. For the purpose of reproducibility, two series of experiments were carried out using the modified template. For each series, the modified template was newly printed (modified 1 and modified 2). The setup of both experiments is shown in Fig. 5.

Statistical analyses were performed using IBM^®^ SPSS^®^ version 29 (IBM Corporation, Armonk, New York, USA) for Windows^®^. The level of significance (α) was set to 0.05.

## Results

**Fig. 6:**
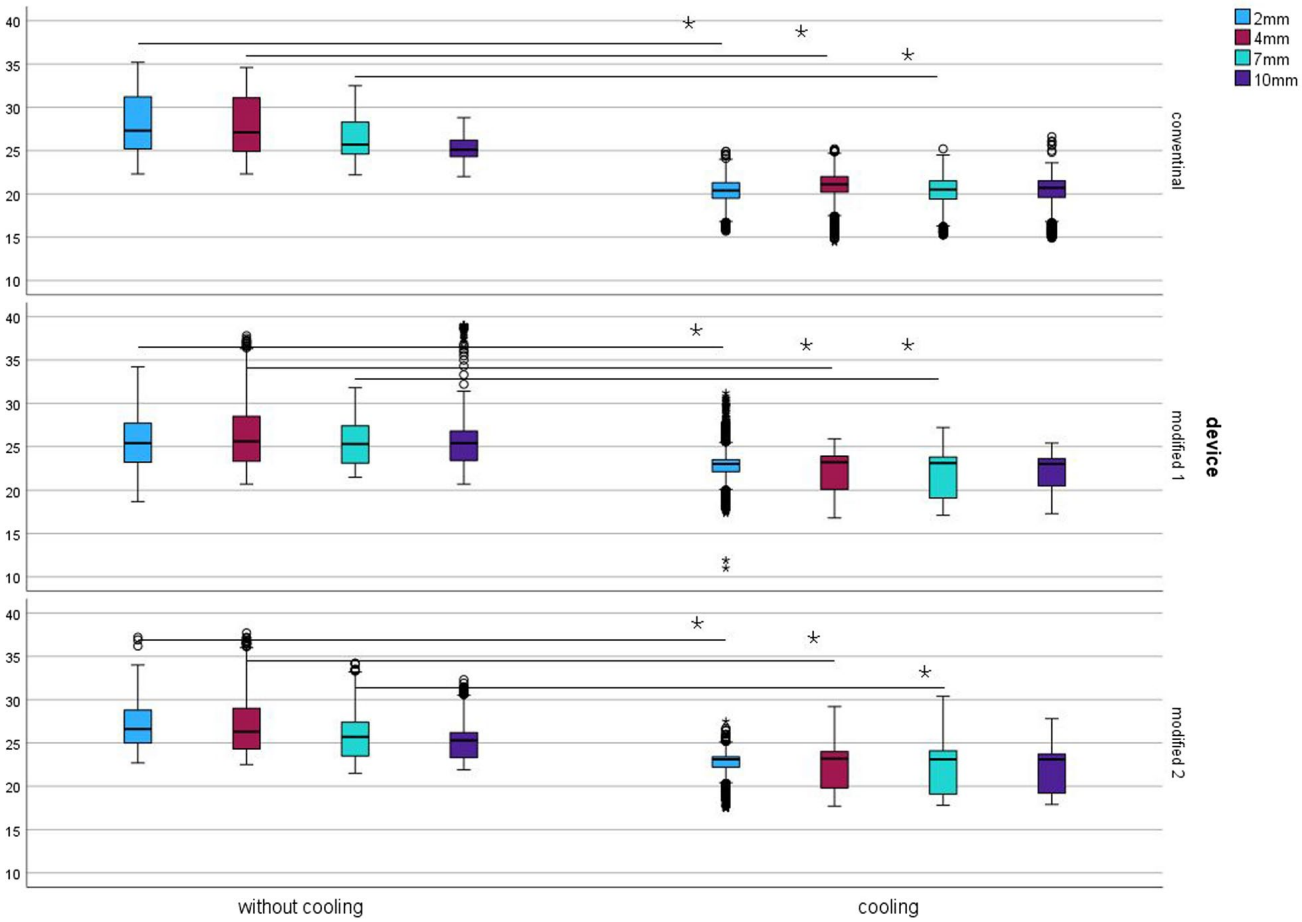
Spread of the measured temperature values (°C) when drilling with and without cooling with conventional (upper boxplots) and modified (middle and lower boxplots) drilling templates; The line with an asterisk indicates the statistically significant difference (p < 0.001); Blue colour depicts the measurements of the thermocouple at a depth of 2 mm in the resin model, red—4 mm, green – 7 mm, and violet—10 mm (y-axis: temperature in °C)

The mean temperature without cooling varied between 25.6 ± 2.8 °C (modified) and 28.1 ± 3.4 °C (conventional); cooling reduced the temperature to 22.5 ± 2.1 °C (modified) and 20.4 ± 1.4 °C (conventional). Figure 6 depicts the boxplot spread of the measured temperature values. At all the measured depths and for all the templates, cooling had a statistically significant (p < 0.001) influence on the mean temperature. ANOVA and Bonferroni correction revealed that at all depths, there was a statistically significant (p < 0.001) difference in cooling across all templates compared to the templates without cooling. However, at a depth of 10 mm, there was not a statistically significant (p = 1.000) difference between the conventional template and the modified 1 template.

The maximum temperatures, depending on the cooling and template conditions, are displayed in Table 1.

**Table 1:** Maximum temperature values (°C) achieved by drilling with and without cooling with conventional and modified drilling templates at depths of 2, 4, 7 and 10 mm

Cooling	Template	2 mm	4 mm	7 mm	10 mm
Without cooling	Conventional	35.2	34.6	32.5	28.8
	Modified 1	34.2	37.8	31.8	39.1
	Modified 2	37.2	37.7	34.2	32.3
With cooling	Conventional	24.9	25.2	25.2	26.6
	Modified 1	31.2	25.9	27.2	25.4
	Modified 2	27.5	29.2	30.4	27.8

## Discussion

The results of the current study reject the research hypothesis, as the modified drilling templates did not produce lower temperatures during drilling than the conventional drilling templates did.

For the drilling experiments, a conventional surgical contra-angle handpiece and a surgical motor were employed, simulating experimental conditions that were as close to the clinical setting as possible. Moreover, it has been reported that the type of surgical motor does not affect the temperature during drilling [10]. Drilling was carried out by a single calibrated operator at a constant pressure of approximately 1 kg. The pressure applied in the current study was derived from data from comparable studies [10, 11]. However, despite calibration, the operator could not produce a constant pressure during the entire drilling process; to accomplish this, an automated and standardized machine-drive test assay would have been required. Nevertheless, the current setup more closely resembles clinical conditions where the pressure produced by the surgeon is also not constant. The force or pressure exerted on the drill is one of the major causative agents for overheating the bone. Bachus et al. reported that the temperature measured during drilling decreases with increasing force because the drilling time decreases with increasing force [12]. Brisman et al. highlighted that increasing either force (in the range of 12–24 N) or speed (1800–2400 rpm) increased the temperature, although the temperature did not increase significantly with increasing parameters [13]. In addition to time and force also sharpness and wear of the drill might affect the heat generation. In order to justify these effects, the drills were used for a maximum of 10 drillings.

Owing to their uniform consistency and equal density, the samples used were printed from synthetic resin, which allowed both template experiments to be carried out under the same conditions. There are various densities of bone (compact and spongy bone) that are encountered at different drilling depths, which present relevant differences from the experimental model. A point of limitation are the complex structures (such as tongue, cheek and flap) that have an influence in everyday clinical practice. In the present laboratory experiment, these structures were neglected due to simplification, as it was a matter of fundamental feasibility. As preliminary experiments have shown, drilling within the synthetic resin with a pilot drill took longer than drilling in natural bone, which might have been due to the density of the synthetic resin. To improve the model, the samples were supplied with a central tunnel to reduce the drilling time. The channel also made it easier for the operator to maintain a constant drilling force of 1 kg. Another limitation of the study was certainly that the resin model cannot reproduce the most heat-regenerated cortical layer of the bone. Although the acrylic model does not allow the complex bone structure to be simulated, it does allow a comparative measurement of the temperatures generated during drilling. Two different approaches for measuring temperature changes have been described in the literature: via thermocouples (as employed in the current study) or infrared thermography systems [14]. In the current setting, the use of an infrared thermography system was not advisable because water pouring over the drilling site during cooling would have distorted the measurements.

The results of the current study revealed a cooling effect for conventional drilling templates when the coolant was dispensed over the template. Thus, the temperatures measured during cooling were lower than those measured without cooling. Nevertheless, the conventional cooling system might lead to an unsatisfactory cooling effect because the coolant was dispersed into the environment rather than directly to the drill and the tooth. Orgev et al. evaluated a surgical drilling template with a cooling system that directed the coolant close to the tooth site but did not measure temperature changes [15]. In a similar study, Tech et al. reported that temperatures in bovine bones remained lower than 47 °C when a cooling system was used that directed the coolant directly to the implantation site. However, no comparisons were made for settings with and without cooling or for conventional and modified cooling systems [16]. The current experimental design allowed us to compare the cooling efficiency of two different drilling templates by measuring the temperature in the drilled shaft in identical mandibular models. However, the results indicated that the modified design for the drilling template did not improve the cooling efficiency compared with that of the conventional cooling system. Thus, it can be assumed that the coolant flowing around the drill, rather than the amount of coolant supplied to the bone penetration site, is responsible for the cooling effect.

## Conclusion

Compared with a conventional cooling system in a standard drilling template, a modified drilling template with an internal cooling channel delivering the coolant directly to the drill did not produce an improved cooling effect.

## Data Availability

No datasets were generated or analysed during the current study.

## Abbreviations

CAD: Computer aided design
CAM: Computer aided manufacturing
CBCT: Cone beam computed tomography
°C: Degrees celsius
DICOM: Digital imaging and communications in medicine
3D: Three-dimensional
kg: Kilogram
mm: Millimetre
mL: Millilitre
p: P value
STL: Standard tessellation language
